# Society Meetings

**Published:** 1912-12

**Authors:** 


### SOCIETY MEETINGS.

---

Secretaries of County Societies of the western four District Branches of the MEDICAL SOCIETY OF THE STATE OF NEW YORK and of other professional organizations in this territory, are requested to send notices and brief abstracts for free publication. Similar notices of societies of general scope are also desired. Full proceedings will be printed at cost. Copy should be received before the 20th of the month to insure insertion in the next issue.

---

THE SCIENTIFIC STUDY OF ALCOHOLIC PROBLEMS. In New York City in 1870, a number of physicians and medical experts formed an organization, called The American Society for the Study of Alcohol and Other Narcotics. This was the first medical association in the world to take up the study of alcohol and inebriety, and determine from clinical experience, laboratory studies, and grouping of facts the phenomena of the spirit and drug neuroses.

The special purpose was to study this subject above all theories and conclusions, particularly its etiological, physiological, therapeutic and medico-legal relations; also to compile and make available such studies for home, office and institutional treatment, and point out the remedies and prophylactic measures for restoration and cure.

The 42nd annual meeting of this society will be held at Washington, D. C., Dec. 10th and 11th, 1912. A large programme of scientific papers from medical men, many of them distinguished foreigners, will be presented. This meeting and the subjects discussed will attract a great deal of attention, not only among medical men, but an increasing number of laymen, who have an intense personal interest in alcohol and drug taking.

Programs and further information can be had by addressing the Secretary, Dr. T. D. Crothers, of Hartford, Conn.

A joint meeting of the New York Neurological Society was held with the Section on Neurology and Psychiatry of the N. Y. Academy of Medicine, November 12. The program:

A case of "Periodicity in the Male," by Dr. C. P. Oberndorf. Aborted Forms and Preparalytic Stages of Acute Poliomyelitis as Observed in the Buffalo Epidemic, by Dr. E. A. Sharp, of Buffalo. Discussion by Professor James Putnam and Dr. Nelson G. Russell of Buffalo. "Experiences with the Meningeal Fibro-Endotheliomata," (twenty minute presentation), by Dr. Harvey Cushing. Discussion by Drs. Sachs, Dana, Starr, Elsberg and Taylor. L. Pierce Clark, M. D., President. E. G. Zabriskie, M. D., Secretary.

---

The Buffalo Medical and Surgical League met Oct. 14, at the Lafayette Hotel. The paper of the evening was: "Empyema in Children," by Dr. A. W. Hengerer. In the discussion, Dr. Reu reported a case of amoebic infection of the gall bladder that had penetrated into the pleural cavity and thence into the lung. This patient expectorated quantities of material containing amboebae.

---

The Buffalo Academy of Medicine has held the following meetings since lated noted: Oct. 22, Pathologic Section: Clean Milk from the Public Health Standpoint, Dr. Charles E. North of New York City, who described his model farm at Homer and advocated the pasteurization of all milk. He showed lantern slides to illustrate the bacterial content of certified milk. Dr. N. G. Russell spoke on Certified Milk in Buffalo and a general discussion followed. Surgical Section, Oct. 29: Dr. Chevalier Jackson of Pittsburg, gave a Lantern Demonstration of Some Advances in the Methods of Examination of the Air and Food Passages, mentioning particularly the advantages of direct laryngoscopy, bronchoscopy for the extraction of foreign bodies, and oesophagoscopy and gastroscopy, the last especially in co-operation with the surgeon during abdominal section. It is typic of the humantarian aim of the medical profession that Dr. Jackson spoke most modestly of his wonderful original work and laid stress, not on the scientific and technical aspects of his subject, so much as on the necessity of legislation to protect little children—and occasionally adults too,—from the action of alkaline caustics sold for domestic use without sufficient cautionary labeling. Medical Section: November 12, Dr. Charles G. Stockton read a paper on Visceral Syphilis, with collation of experience especially for the stomach and liver, with a view to establishing diagnostic standards. Dr. A. L. Benedict reported a case of

Elephantiasis and Ascites due to Obstruction at the Receptaculum Chyli or in the Thoracic Duct, with allusion to a case of terminal left sided anasarca probably due to coagulum obstructing the major duct.

Obstetric Section, November 19. Dr. E Gustave Zinke, of Cleveland, discussed The Comparative Merits of Medical and Surgical Treatment of Eclampsia, strongly favoring the use of large doses of veratrum viride, mild purgation, sweating and milk diet.

---

The Medical Society of the County of Niagara met at Lockport, Nov. 12. Dr. Scott of Niagara Falls read a paper on "The Art of Differentiating Suicide from Homicide."

---

The Rochester Academy of Medicine met Nov. 13 and listened to a paper by Dr. F. J. Parmenter of Buffalo, on "The Relation of Vaccine Therapy to Medicine and Surgery."

The Joint Medical Societies of Rochester, gave a dinner on Nov. 21 to Prof. Von Noorden and listened to his paper on "The Treatment of Chronic Nephritis."

The Joint Medical Societies of Rochester, had another dinner and meeting on Nov. 25, in honor of Hon. Homer Folks, Dr. A. H. Garvin of Raybrook, and Dr. J. H. Pryor of Buffalo.

